# Impact of Preexisting Alcohol Use Disorder, Bipolar Disorder, and Schizophrenia on Ischemic Stroke Risk and Severity: A Lebanese Case-Control Study

**DOI:** 10.3390/healthcare11040538

**Published:** 2023-02-11

**Authors:** Elise Maalouf, Souheil Hallit, Pascale Salameh, Hassan Hosseini

**Affiliations:** 1 Life and Health Sciences Department, Paris-Est University, 94000 Créteil, France; 2 School of Medicine and Medical Sciences, Holy Spirit University of Kaslik, Jounieh P.O. Box 446, Lebanon; 3 Applied Science Research Center, Applied Science Private University, Amman 11931, Jordan; 4 Research Department, Psychiatric Hospital of the Cross, Jal Eddib P.O. Box 60096, Lebanon; 5 School of Medicine, Lebanese American University, Byblos 5053, Lebanon; 6 INSPECT-LB: Institut National de Santé Publique, Épidémiologie Clinique et Toxicologie-Liban, Beirut 1103, Lebanon; 7 Medical School, University of Nicosia, Nicosia 2417, Cyprus; 8 Faculty of Pharmacy, Lebanese University, Hadat 1533, Lebanon; 9 UPE-C, Faculté de Santé, Université Paris-Est Créteil, INSERM U955-E01, IMRB, 94000 Creteil, France; 10 Hopital Henri Mondor, APHP, 94000 Créteil, France

**Keywords:** ischemic stroke, alcohol use disorders, bipolar disorders, schizophrenia, stroke severity

## Abstract

Background: Stroke remains a major leading cause of morbidity and death globally. For ischemic stroke, the most frequent type of stroke, there are numerous risk models and risk assessments offered. Further research into potential risk factors or triggers is being sought to improve stroke risk models. Schizophrenia, bipolar disorder, and alcohol use disorder are all common causes of serious mental illnesses in the general population. Due to the tangled relationship between stroke and many chronic illnesses, lifestyle factors, and diet that may be present in a patient with a mental disease, the relationship between mental diseases and stroke requires further validation. Consequently, the purpose of this study is to assess the potential influence of bipolar disorder, schizophrenia, and alcohol use disorder on stroke patients as compared to non-stroke participants, after controlling for demographic, physical, and medical conditions. We aimed, as a secondary objective, to evaluate the impact of these pre-existing disorders on stroke severity levels. Methods: This research is a case-control survey study involving 113 Lebanese patients with a clinical diagnosis of ischemic stroke and 451 gender-matched volunteers without clinical signs of stroke as controls recruited from several hospitals in Lebanon (April 2020–April 2021). Based on the participant’s consent, data was collected by filling out an anonymous paper-based questionnaire. Results: All of the odds ratios (ORs) generated by our regression model were greater than 1, indicating that the factors studied were associated with an increased risk of ischemic stroke. As such having schizophrenia (adjusted OR [aOR]: 6.162, 95% confidence interval [CI]: 1.136–33.423), bipolar disorder (aOR: 4.653, 95% CI: 1.214–17.834), alcohol use disorder (aOR: 3.918, 95% CI: 1.584–9.689), atrial fibrillation (aOR: 2.415, 95% CI: 1.235–4.721), diabetes (aOR: 1.865, 95% CI: 1.117–3.115), heart diseases (aOR: 9.890, 95% CI: 5.099–19.184), and asthma-COPD (aOR: 1.971, 95% CI: 1.190–3.263) were all involved with a high risk of developing an ischemic stroke. Moreover, obesity (aOR: 1.732, 95% CI: 1.049–2.861) and vigorous physical activity (aOR: 4.614, 95% CI: 2.669–7.978) were also linked to an increased risk of stroke. Moreover, our multinomial regression model revealed that the odds of moderate to severe/severe stroke were significantly higher in people with pre-stroke alcohol use disorder (aOR: 1.719, 95% CI: 1.385–2.133), bipolar disorder (aOR: 1.656, 95% CI: 1.281–2.141), and schizophrenia (aOR: 6.884, 95% CI: 3.294–11.492) compared to people who had never had a stroke. Conclusion: The findings in our study suggest that individuals with schizophrenia, bipolar disorder, and alcohol use disorder may be at a higher risk for ischemic stroke and exhibit more severe symptoms. We believe that the first step toward creating beneficial preventative and treatment interventions is determining individuals with schizophrenia, bipolar disorder, or alcohol use disorder, assessing their risk of ischemic stroke, developing more integrated treatments, and closely monitoring the long-term outcome in the event of an ischemic stroke.

## 1. Background

### 1.1. Ischemic Stroke: Etiology and Disease Burden

Despite ongoing research into novel acute predictors or triggers, stroke remains a major leading cause of morbidity and death globally [1]. In 87% of all strokes, a blood vessel supplying blood to the brain is blocked. These are alluded to as “ischemic strokes” [2]. Ischemic stroke occurs when the blood supply to the brain is interrupted, usually due to a thrombotic or embolic event. The blood vessel itself becomes dysfunctional in a thrombotic event, blocking blood flow to the brain. This reduces the oxygen and nutrient supply to the brain, resulting in tissue damage. This may be caused by atherosclerosis, arterial dissection, fibromuscular dysplasia, or inflammation. An embolic event occurs when debris from another part of the body, most commonly the heart and large arteries of the upper chest and neck, enters the bloodstream and travels through the brain’s blood vessels, eventually blocking blood flow in the process. Atrial fibrillation, a condition characterized by an irregular heartbeat, is the leading cause of embolism. It increases the likelihood of blood clots forming in the heart, rupturing, and traveling to the brain [2,3,4]. For ischemic stroke, the most frequent type of stroke, there are numerous risk models and risk assessments offered. Nevertheless, they only cover a well-established list of risk factors, and these variables might not always predict all ischemic strokes. As a result, further research into potential risk factors or triggers is being sought with the goal of improving the simulation process of stroke risk models and lowering stroke risks. 

### 1.2. Overview of Bipolar Disease, Schizophrenia, and Alcohol Use Disorder

Schizophrenia, bipolar disorder (BD), and alcohol use disorder (AUD) are all common causes of serious mental illnesses in the general population. To begin, both BD and schizophrenia are mental disorders that share certain characteristics but have substantial differences. The American Psychiatric Association’s Diagnostic and Statistical Manual of Mental Disorders (DSM-5) defines BD as a group of brain disorders that cause extreme fluctuations in a person’s mood, energy, and ability to function [5]. Schizophrenia is defined by the DSM criteria as a severe, chronic mental disorder characterized by disturbances in thought, perception, and behavior, specifically a loss of touch with reality, dis-organized speech, thinking difficulties, and a lack of motivation [5,6]. Following that, the DSM-5 categorizes AUD as a substance use disorder; indeed, a person with AUD has a decreased capacity to cease or regulate alcohol consumption despite unfavorable social, occupational, or health consequences. Alcohol is frequently consumed in larger amounts or for a longer period than anticipated [5].

### 1.3. Studies Linking These Diseases to an Increased Ischemic Stroke Risk

According to research, psychiatric patients had a considerably higher risk of death compared with individuals who had no episodes of treatment for a mental disorder, regardless of the cause of death [7,8,9]. This is particularly the case for patients suffering from severe mental illnesses, such as schizophrenia [10,11] and BD [12]. Patients with substance abuse problems have been shown to be at the greatest risk [7,13]. In addition to unnatural deaths (including both accidents and suicides), cardiovascular, cerebrovascular, and respiratory disorders are the primary causes of increased mortality in mental patients [14].

Several studies have been conducted over the last decade to investigate the risk of cardiovascular and respiratory diseases among psychiatric patients [15,16,17,18,19,20], but within this specific population, little emphasis has been placed on any scrutiny of the risk of developing cerebrovascular diseases. Descriptions of the existence of cerebrovascular diseases in individuals with a prior psychiatric illness are less common, and studies that specifically highlight silent cerebrovascular diseases, as a finding in patients with a variety of mental problems are extremely rare. Indeed, evidence in the literature that psychiatric disorders, such as BD and schizophrenia, may acutely increase the risk of stroke was deemed controversial since numerous studies have shown that these mental diseases coexist with common chronic diseases, such as hypertension, diabetes, hyperlipidemia, and obesity, all of which are risk factors for cerebrovascular disease [21,22]. Many prospective studies have been carried out over the last few decades to assess the relationship between BD, schizophrenia, and stroke risk [23,24,25], but due to the tangled relationship between stroke and many chronic illnesses, lifestyle factors, and diet that may be present in a patient with mental disease, the relationship between mental diseases and stroke requires further validation. Additionally, in the case of alcohol use disorder, alcohol consumption or alcoholism [26] was evaluated more without previously investigating the addictive use of alcohol and the risk of ischemic stroke until recently, as one study covered this subject in geriatric patients with prediabetes only [27].

A case-control study with 2063 young adults between the ages of 18 and 45 who had an ischemic stroke and 8252 age- and sex-matched controls found that patients who had an ischemic stroke at an age younger than 45 were more likely to have a history of BD, unipolar depression, anxiety disorders, and AUD than those who did not [21]. Prospective data, for instance, revealed that within the first five years after hospitalization, young patients with schizophrenia had a twofold higher risk of stroke compared to the control group [22]. Furthermore, a modest but statistically significant positive link between schizophrenia and the risk of stroke incidence and mortality was confirmed in a meta-analysis of six cohort studies [28]. Moreover, Wu et al. found that throughout a seven-year follow-up period, individuals with BD had a higher risk of developing a stroke than controls [29]. In contrast, a Danish registry dataset was used in a study to estimate the risk among patients previously discharged with an affective disorder prior to receiving a stroke diagnosis. The researchers discovered no link between manic or bipolar disorder and stroke [30]. Following that, in a meta-analysis of research that reviewed the association between alcohol consumption and stroke, heavy alcohol use had been reported to increase the relative risk of stroke [23]. A recent study found that AUD in elderly prediabetes patients increased the risk of stroke by up to 33%, but these findings may be limited in their generalizability [27]. As a result, few studies have specifically investigated a link between these psychiatric conditions and ischemic stroke.

### 1.4. Studies Linking These Diseases to an Increased Ischemic Stroke Severity

Despite the fact that numerous risk factor models have been developed in the attempt to explain the nexus relationship between stroke and mental diseases by assessing shared risk factors or pathological mechanisms and determining causality, it has been shown that pre-existing affective disorders predict more severe strokes on admission and negatively affect functional and cognitive outcomes after stroke [31,32,33]. These findings indicate that mental health should not be overlooked when evaluating the likelihood of stroke, as BD and schizophrenia may increase the risk of stroke and worsen recovery from a stroke event. In addition, the evidence for a potential causal link between AUD and stroke remains inconclusive. In the available studies, only the relationship between alcohol consumption and stroke severity was evaluated [34,35]; thus, AUD-related stroke severity was not examined. Even with the current evidence, much remains to be validated regarding the relationship between AUD, schizophrenia, BD, and stroke severity.

### 1.5. Rationale and Objectives of the Study

Stroke prevalence and severity have surged in Lebanon over the last decade, and stroke has now become the country’s second leading cause of mortality. Further, Lebanon’s stroke prevalence may be greater than that of other developing countries in the region [36,37]. Hence, identifying the characteristics associated with ischemic stroke by attempting to address the presence of a prior psychiatric disease, as has been suggested for other clinical conditions [38,39,40,41], could be a crucial step towards reducing the burden of stroke in the Lebanese population. 

It is well known that post-stroke psychosocial consequences affect quality of life, functional recovery, cognitive function, and health care use in stroke survivors. Additionally, specific mental diseases, such as affective disorders, in particular schizophrenia and BD, have been mostly studied with an emphasis on assessing general stroke risk in a specific population, primarily young individuals, or their impact on recovery or long-term prognosis following a stroke [22,29]. However, whether these aforementioned diseases increase stroke incidence independently or whether this relationship is confounded by the population’s prevalent stroke risk factors, such as unhealthy lifestyles, whether or not pursuing a diet rich in healthy foods, such as whole grains, fruits, vegetables, seafood, beans, and nuts, and obesity remains unknown. As a result, the natural history of mental disease as a stroke risk factor is still fraught with controversy. Moreover, when assessing the risk of ischemic stroke in a population with a history of psychiatric disorders, a broad perspective on past medical history, lifestyle, and diet is uncommon. 

To date, no research has been conducted in Lebanon assessing the link between AUD, BD, schizophrenia, and ischemic stroke risk and severity. Furthermore, in general, this topic is frequently overlooked in research, with a limited number of research surveys. Therefore, additional research is required to investigate and confirm whether these mental disorders are associated with an increased risk of stroke and increased stroke severity. Consequently, the purpose of this study is to assess the potential influence of BD, schizophrenia, and AUD on stroke patients as compared to non-stroke participants, after controlling for demographic, physical, and medical conditions. We aimed, as a secondary objective, to evaluate the impact of these pre-existing disorders on stroke severity levels.

## 2. Methods

### 2.1. Study Design

This is a case-control survey study aiming to determine the potential influence of BD, schizophrenia, and AUD on stroke patients and to evaluate their impact on stroke severity levels as compared to non-stroke participants. Cases were Lebanese individuals aged 18 years and above who received a computed tomography (CT) and/or magnetic resonance imaging (MRI) confirming the clinical diagnosis of ischemic stroke at hospitals and rehabilitation facilities in Beirut and Mount Lebanon. Indeed, CT scans and MRIs are two of the most effective stroke screening tests. This is due to the fact that these imaging tests generate an exact image of the head, including the tissue and blood vessels. Both employ X-rays and computers to create a series of images of the inside of the head. An MRI can detect any brain injury within an hour of the onset of stroke symptoms, as opposed to a CT scan, which can require several hours to reveal any blood flow obstructions [42]. The American Stroke Association classifies a stroke as ischemic whenever brain imaging demonstrates an acute infarction or no signs of bleeding [43]. The physician’s diagnostic and imaging findings, which were all recorded in the patient’s file, were used to support the ischemic stroke diagnosis. Cases were excluded due to a lack of written consent, clinical information, a CT/MRI report, or patients diagnosed with hemorrhagic stroke. 

During the research period, we identified four volunteers for each case, gender-matched, without clinical signs of stroke (confirmed by a CT scan) or a history of stroke, who were included as controls. The controls were chosen from either the same hospital as the cases or from the community. Among the hospital-based sources were individuals hospitalized or visiting outpatient clinics for diseases or treatments unrelated to stroke or transient ischemic attack, as well as visitors or relatives of inpatients. Controls were not included if they did not provide written approval for the survey to be undertaken. 

**Figure 1:**
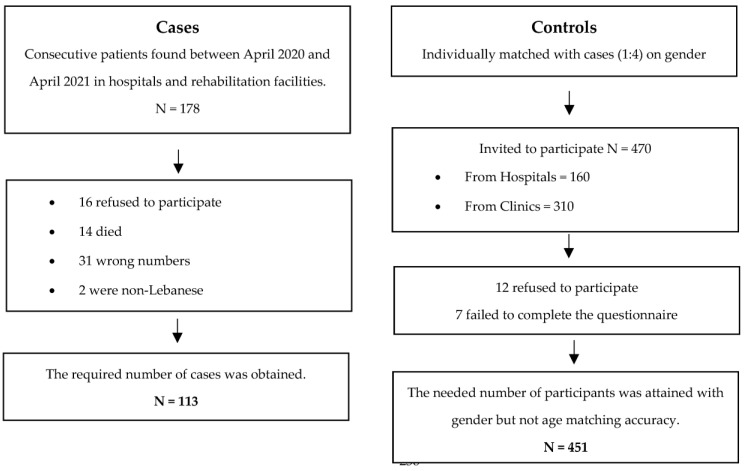
Participants’ flowchart.

The participants were given a consent form that explained the purpose of the study, the advantages, the concerns, and the confidentiality of the data collected. The study’s participation was completely voluntary, with no financial inducement. The data was gathered between April 2020 and April 2021 by filling an anonymous paper-based questionnaire contingent on the participant’s choice. The questionnaire, as with the case and control groups, was completed in a face-to-face interview. The same methodology has been used in a previous paper [44]. Details of this method are presented in Figure 1.

### 2.2. Minimal Sample Size Calculation

With a margin of error of 5%, a power of 80%, and an allocation number of 1:4, the Epi info program generated a minimal sample of 564 participants (113 Lebanese patients with ischemic stroke and 451 controls), to allow for appropriate bivariate and multivariable analysis. It was based on the number of controls exposed to mental health problems estimated to be 17% [45], and the OR of stroke within 30 days of a hospital visit for a mental health concern, reported to be 3.11 [46] (the OR is a bit high, resulting in a small, calculated sample size; so, OR = 2).

### 2.3. Questionnaire and Variables

The questionnaire was written in Arabic, Lebanon’s native language, and took around 60 min to complete. The questionnaire assessed participants’ demographic characteristics, such as age, gender, and marital status, as well as social factors including region, level of education, occupation, and monthly income, and health queries such as personal history of hypertension, hyperlipidemia, diabetes, cardiovascular disease, atrial fibrillation, cancer, stroke type, date of stroke, and severity, using the NIHSS score, which measures multiple aspects of brain function, including consciousness, vision, sensation, movement, speech, and language, and ranges from 0 to 42. The levels of stroke severity, as measured by the NIHSS scoring system, were categorized as follows: no stroke symptoms: 0; minor stroke: 1–4; moderate stroke: 5–15; moderate to severe stroke: 16–20; and severe stroke: 21–42 [47]. 

Moreover, patients’ lifestyle choices, especially:

- maintaining a healthy body weight by assessing the body mass index (BMI),
- following a healthy diet, as measured by the Mediterranean diet (MeD) adherence:

A 14-item validated tool for measuring adherence to a high-quality dietary pattern (the Mediterranean diet) [48]. A higher score indicates greater adherence to a Mediterranean-style diet (Cronbach’s α in this study = 0.801),

- being physically active by evaluating the international physical activity questionnaire (IPAQ)–short version:

The international physical activity questionnaire (IPAQ) is a 7-item questionnaire designed to assess health-related physical activity (PA) in populations in the past 7 days [49]. The IPAQ total score was calculated in PA metabolic equivalent of task (MET)-minutes per day or week. This study’s data processing and analysis were computed in accordance with the standard IPAQ scoring procedure [50]. The total weekly PA MET-minutes were obtained by summing the MET-minutes calculated for each PA intensity level (moderate intensity = 4.0 MET, vigorous intensity = 8.0 MET, and walking = 3.3 MET). The stated sitting time was determined as per weekday. A high level of physical activity on the IPAQ indicates that the physical activity levels correspond to at least one hour of moderate-intensity activity per day or more. In this study, Cronbach’s alpha was 0.726.

Preexisting psychiatric disorders prior to the ischemic stroke or enrollment at the time of hospitalization for stroke were verified by our questionnaire for all cases, even if they were mentioned in their clinical file at admission, and assessed for patients unable to communicate adequately; indeed, their guardians, who had been present all the time with them and were aware of their psychological states, responded to the scales below; as for the controls, they were interviewed and checked for previous psychiatric diagnoses, including AUD, BD, and schizophrenia.

▪ The alcohol use disorder (AUD) was evaluated using the **alcohol use disorder identification test (AUDIT)**. This is a 10-item validated questionnaire developed by the World Health Organization (WHO) to assess alcohol consumption, drinking behaviors, and alcohol-related problems defined as risky or hazardous consumption or any AUD [51]. A score of 8 or higher indicates risky or harmful alcohol use [52] (Cronbach’s α in this study = 0.733).
▪ The BD was assessed using the **mood disorder questionnaire (MDQ)**. The MDQ is a 15-item self-report screening instrument that is most sensitive to bipolar I (depression and mania) disorder and less sensitive to bipolar II (depression and hypomania), or bipolar not otherwise specified (NOS) disorder [53]. It has 13 yes/no questions about bipolar symptoms and two more about symptom co-occurrence and impaired functioning. A total score is calculated for questions 1–13, with “Yes” providing a score of 1 and “No” providing a score of 0. The traditional scoring method for reaching the BD threshold is as follows:
○ A score of 7 or higher on questions 1–13 AND,
○ Select “yes” for the item requesting whether the symptoms occurred at the same time (Question 14) AND,
○ Symptoms caused either “moderate” or “serious” problems (Question 15),
○ (Cronbach’s α in this study = 0.887).
▪ Schizophrenia was assessed using the **mini international neuropsychiatric interview (MINI) for psychotic disorders** studies for the DSM-5 diagnostic criteria for schizophrenia [54]. Questions are phrased to allow only “yes” or “no” answers. It describes the five major symptoms of psychotic disorders: (1) delusions, (2) hallucinations, (3) disorganized speech, (4) disorganized or catatonic behavior, and (5) negative symptoms. Schizophrenia diagnosis requires the detection of two (or more) of the aforementioned symptoms, each of which must be present for a significant portion of time during a one-month period (or less if successfully treated), with at least one of them being (1), (2), or (3).

### 2.4. Translation Procedure

The scales that were not validated in Lebanon were translated from English to Arabic. They were prepared by two experts: one who is familiar with the scales’ vocabulary, speaks Arabic as a native language, and is fluent in English, and another who is fluent in Arabic and speaks English as a first language. At the end of the procedure, experts compared the two English versions to see whether there were any discrepancies (Available in English in the Appendix A).

### 2.5. Statistical Analysis 

The SPSS version 23 was used to perform the statistical analyses in this study, with descriptive analyses being carried out on all the identified variables, including the frequencies (percentages) and means (standard deviations). A computation of the skewness and kurtosis demonstrated the normality of the distribution, including all scales; values for asymmetry and kurtosis between −1 and +1 were deemed acceptable in order to confirm the normal univariate distribution [55]. A bivariate analysis was then used to identify possible risk factors for ischemic stroke. To compare percentages between two groups, the chi-square and Fisher exact tests were used. Student’s test was applied to compare means between two groups since all scales had a normal distribution. A *p*-value of less than 0.05 was regarded as significant. 

In the logistic regression models, all covariates with a *p*-value of less than 0.2 in the bivariate analysis were included. A backward stepwise logistic regression analysis was used to investigate the odds ratio (OR) with a 95% CI of AUD, BD, and schizophrenia among subjects with ischemic stroke and the control group. The omnibus test was supposed to be significant to indicate that at least one of the introduced covariates significantly affects the dependent variable. The Hosmer–Lemeshow test was supposed to be non-significant in order to demonstrate the test’s adequacy. The CI was set at 95%, and a *p*-value less than 0.05 was considered significant.

The levels of stroke severity, as measured by the NIHSS scoring system, were taken as a dependent variable in a multinomial logistic regression. The chi-square test was employed to compare categorical variables, whereas the ANOVA test was used to compare three means. All variables with *p* < 0.2 were included as independent variables in the final model. The significance level was set at *p* < 0.05.

## 3. Results

### 3.1. Effect of the Variables on Ischemic Stroke Risk

#### 3.1.1. Demographic Data of Patients with Ischemic Stroke and the Control Group (N = 564)

Table 1 outlines the demographic factors (previously shown in a prior study [44]). When compared to non-ischemic stroke participants, ischemic stroke patients had a significantly higher mean age (65.5 vs. 62.9) and a greater percentage of married individuals (75.2% vs. 63.4%). In addition, ischemic stroke patients had a significantly lower educational level but a higher monthly income than controls, according to the data. Furthermore, preexisting physical comorbidities, such as hypertension (72.6% vs. 56.3%, *p* = 0.002), dyslipidemia (57.5% vs. 45.5%, *p* = 0.027), diabetes (36.3% vs. 26.6%, *p* = 0.048), heart diseases (42.5% vs. 11.8%, *p* < 0.001), atrial fibrillation (30.1% vs. 8.0%, *p* < 0.001), asthma-COPD (38.9% vs. 26.2%, *p* = 0.008), and obesity (61.9% vs. 50.8%, *p* = 0.035) were more prevalent in participants with ischemic stroke than in those without. Interestingly, non-ischemic participants were found to be more likely than ischemic stroke patients to adhere to a MeD (7.6 vs. 5.4, *p* < 0.001) and to engage in more moderate to vigorous physical activity (163.83 vs. 79.28, *p* < 0.001).

#### 3.1.2. Bivariate Analysis of Other Factors Associated with Ischemic Stroke

Our observations imply that ischemic stroke patients had considerably higher incidences of prior AUD, BD, and schizophrenia than controls (Table 2).

#### 3.1.3. Multivariable Analysis: Logistic Regression

All significant parameters from the bivariate analysis were included in the multivariable logistic regression. The model was suitable, and the Hosmer–Lemeshow test was adequate. 

Our regression model revealed that having schizophrenia (adjusted odds ratio [aOR]: 6.162, 95% confidence interval [CI]: 1.136–33.423), BD (aOR: 4.653, 95% CI: 1.214–17.834), AUD (aOR: 3.918, 95% CI: 1.584–9.689), atrial fibrillation (aOR: 2.415, 95% CI: 1.235–4.721), diabetes (aOR: 1.865, 95% CI: 1.117–3.115), heart diseases (aOR: 9.890, 95% CI: 5.099–19.184), and asthma-COPD (aOR: 1.971, 95% CI: 1.190–3.263) were all involved with a high risk of developing an ischemic stroke (Table 3). Moreover, obesity (aOR: 1.732, 95% CI: 1.049–2.861) and vigorous physical activity (aOR: 4.614, 95% CI: 2.669–7.978) were also linked to an increased risk of stroke.

**Figure 2:**
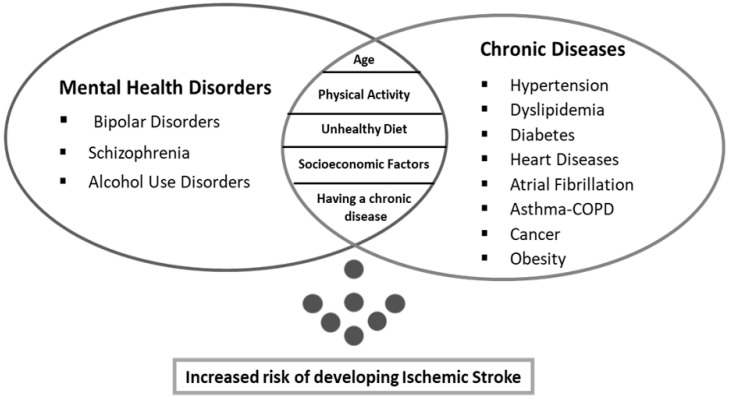
Common risk factors for chronic diseases and mental disorders, as well as their impact on the risk of an ischemic stroke. Figure summarizing the purpose and findings of our research.

The observations regarding the effect of the Variables on the Risk of Ischemic Stroke are illustrated in Figure 2.

### 3.2. Effect of the Variables on Stroke Severity

#### 3.2.1. Bivariate Analysis of the Variables Associated with the Levels of Stroke Severity

Significantly more married participants had a minor/moderate stroke, while those with a moderate to severe/severe stroke had a lower level of education. In moderate to severe/severe stroke patients, preexisting physical disorders, such as hypertension, dyslipidemia, heart disease, atrial fibrillation, and asthma-COPD were significantly more prevalent. In comparison to those with a minor/moderate stroke or a moderate to severe/severe stroke, those without a stroke have a greater adherence to the Mediterranean diet. When compared to those with no stroke or minor/moderate stroke severity, people with moderate to severe/severe stroke had a higher mean age, a higher mean of AUD and BD, and engaged in more moderate to vigorous physical activity. Schizophrenia was common among patients with moderate to severe/severe stroke. There was no significant association between the severity of a stroke and gender, diabetes, cancer, or obesity (Table 4).

#### 3.2.2. Multivariable Analysis: Multinomial Regression

Our regression analysis showed that having a low level of education versus a high level (aOR:0.130, 95% CI: 0.044–0.383), an AUD (aOR: 1.466, 95% CI: 1.260–1.706), a BD (aOR: 1.229, 95% CI: 1.066–1.416), hypertension (aOR: 2.380, 95% CI: 1.055–5.372), dyslipidemia (aOR: 2.220, 95% CI: 1.024–4.811), heart diseases (aOR: 3.726, 95% CI: 1.396–9.950) were significantly associated with an increased likelihood of having a minor/moderate stroke compared to people who had never had a stroke. However, following a Mediterranean diet (aOR: 0.711, 95% CI: 0.620–0.816) was found to reduce the risk of having a minor/moderate stroke compared to individuals who had never had a stroke (Table 5: Model 1). 

The odds of a moderate to severe/severe stroke increased significantly with age (aOR: 1.321, 95% CI: 1.173–1.488), AUD (aOR: 1.719, 95% CI: 1.385–2.133), BD (aOR: 1.656, 95% CI: 1.281–2.141), schizophrenia (aOR: 6.884, 95% CI: 3.294–11.492), and heart diseases (aOR: 6.486, 95% CI: 1.955–21.525) compared to people who had never had a stroke. Nevertheless, compared to those who had never had a stroke previously, those who adhered to a Mediterranean diet (aOR: 0.573, 95% CI: 0.470–0.698) had a lower risk of suffering a moderate to severe/severe stroke (Table 5: Model 2).

## 4. Discussion

In the present study, examining the relationship between affective disorders, such as schizophrenia, BD, and AUD, and the risk of having an ischemic stroke, individuals who had an ischemic stroke were more likely to have these preexisting affective disorders than the control group. In addition, after adjusting for stroke risk factors, we found that patients with these preexisting disorders have a significantly increased risk of stroke. Alternatively, the severity of a stroke was found to be strongly associated with a history of schizophrenia, BD, and AUD, which was another important finding of this study.

When compared to the general population, people with schizophrenia may have a higher incidence of co-occurring disorders [56]. There is little data available on the prevalence of cerebrovascular diseases in schizophrenic patients. Moreover, during a thorough review of the literature, the evidence to date on this topic is conflicting. According to research, the cerebrovascular risk in schizophrenia patients is higher [24,57,58], lower [59], or similar to the general population [60]. As shown by the current results of our analysis, there is a statistically significant positive relationship between schizophrenia and the likelihood of having an ischemic stroke, which is consistent with previous findings [24]. Indeed, the authors discovered that in the five years following an acute worsening of their baseline, schizophrenic patients younger than 45 years had a twofold increased risk of stroke compared to those without schizophrenia who had been hospitalized for an appendectomy [24]. This risk was much higher in women than in men [24]. Furthermore, individuals with chronic schizophrenia were shown to be at increased risk of stroke in a 2016 study conducted in China [61]. Old age, female sex, smoking history, combination therapy with a range of medicines, high dosages, obesity, and high blood pressure were all linked to stroke in chronic schizophrenia patients [61]. In our study, however, we were able to confirm the presence of a link other than the coexistence of clinical comorbidities as a link in the relationship between schizophrenia and stroke. As a result, a potential pathophysiological mechanism underlying the increased number of subsequent stroke events in schizophrenic patients is reinforced.

Our research revealed that individuals who experienced an ischemic stroke were more likely to have suffered from BD prior to the onset of the stroke. Therefore, a statistically significant association was found between BD and the likelihood of experiencing an ischemic stroke. There is little evidence on the risk of developing stroke in patients with BD, despite the fact that cerebrovascular diseases have been recognized as one of the leading causes of mortality in this patient group [62,63,64]. Nevertheless, some studies have found a link between BD and ischemic stroke, which agrees with our data [23,25]. In fact, in a 2007 study, researchers found that the risk of developing a stroke during the six-year follow-up period was twice as high in individuals with BD as in those who had undergone an appendectomy [25]. These findings were similar to another study published in 2013 that identified patients with BD as having a considerably increased risk of stroke and post-stroke mortality [23]. Previous research has found that people with BD are more likely to have diabetes, hypertension, hypercholesterolemia, and heart disease [65], emphasizing the close relationship between BD and stroke. Upon controlling for these diseases, we were able to prove the existence of a link other than the coexistence of clinical comorbidities in the relationship between schizophrenia and stroke, which supports the hypothesis of a possible pathophysiological mechanism and allows us to consider BD as a modifiable risk factor.

Few studies have examined the relationship between AUD and stroke. In our study, we observed a statistically significant connection between AUD and the occurrence of ischemic stroke. This result was in line with a recent study showing that AUD increased the risk of stroke by up to 33% in elderly prediabetic patients [27]. The majority of epidemiologic studies on the effect of alcohol use on the risk of stroke were conducted with inconclusive results. For instance, a meta-analysis study discovered a J-shaped relationship between alcohol intake and the relative risk of total and ischemic stroke after retrieving 122 relevant reports and 35 observational studies (cohort or case control) with total stroke, ischemic stroke, or hemorrhagic (intracerebral or total) stroke as an end point [26]. Furthermore, moderate alcohol consumption was associated with a lower relative risk of total and ischemic stroke, whereas heavy alcohol consumption was associated with a higher relative risk of total, ischemic, and hemorrhagic stroke [26].

When we extrapolated our findings from other research, we noticed a scarcity in the actual mechanisms that contribute to the association between these mental illnesses and the eventual development of ischemic stroke. This relationship is thought to involve a variety of mechanisms, including an unhealthy lifestyle, comorbid medical conditions (alcohol-induced hypertension, cardiomyopathy, coagulation disorders, atrial fibrillation...) [66,67,68], and the use of psychotropic medications [69,70], all of which may contribute to an increased risk of stroke among patients with the aforementioned mental illnesses; however, they may also be at an increased risk of stroke due to their pre-existing brain vulnerability [71,72,73,74,75], which could explain the findings in our study that led us to consider schizophrenia, BD, and AUD as modifiable risk factors for stroke.

In terms of obesity, atrial fibrillation, diabetes, heart diseases, asthma, and COPD [76,77,78,79], we observed the same link and strength as many other studies and were considered major predisposing factors for stroke occurrence. Although it has been shown that regular physical activity reduces the risk of cardiovascular disease and stroke, the effect of vigorous physical activity per week is unknown. In our study, we discovered that engaging in a high level of physical activity per week may actually increase the risk of an ischemic stroke. High-intensity and prolonged efforts, according to the findings of a study, may increase the risk of death from a heart attack or stroke in people who already have heart disease [80]. Another study found that 1 h of moderate or vigorous physical activity increased the risk of stroke by 2.3 times, whereas habitual physical activity significantly reduced the risk of stroke [81].

Moreover, pre-stroke AUD, BD, and schizophrenia were significant risk factors for stroke severity, thereby increasing an individual’s susceptibility to the disease. Indeed, pre-existing affective disorders, particularly schizophrenia and BD, have been shown to predict more severe strokes on admission and to have a negative impact on functional and cognitive outcomes following stroke [31]. Additionally, a large Swedish study found that patients with pre-stroke psychosis had a worse prognosis and were less inclined to seek secondary pharmacological prevention after stroke [32]. Furthermore, a recent Scottish study discovered that having a severe mental illness, such as schizophrenia or BD, was linked to an increased risk of mortality and further vascular events [33]. Consistent with these studies, our findings provide corroborated evidence of an association between pre-stroke schizophrenia, BD, and stroke severity. At last, the potential role of AUD in stroke development is understudied, with very few studies investigating its relationship with stroke severity or outcome after stroke and with the majority of research focusing on heavy or moderate alcohol consumption without considering the underlying psychological disease. Nevertheless, our findings suggest that AUD may be a significant risk factor for stroke severity, warranting more research into the role of AUD in stroke development and outcomes. Numerous mechanisms could be proposed to explain this potential association, such as the induction of cardiac arrhythmias and cardiac wall motion abnormalities that predispose to cerebral embolism, the induction of hypertension and vascular inflammation, [82,83]. Furthermore, epidemiological evidence suggests that drinking alcohol regularly is linked to a higher risk of stroke-related mortality [35].

## 5. Limitations and Strengths

The case-control approach of this study limits its outcomes. Therefore, information on earlier exposures acquired by questioning study participants is prone to memory bias, particularly when dealing with patients with severe stroke complications and seeking to obtain personal information from their guardian. Furthermore, we lacked prescription data on psychiatric drugs, such as antipsychotics and mood stabilizers, which have been known to be associated with stroke risk, as well as possible drug-drug interactions, which may be confounding factors that could have affected our findings. We also had little information on the duration and compliance with current psychopharmacotherapy, as well as comprehensive data on past hospitalizations for psychiatric disorders, which would have been useful in demonstrating an association between the time of the last psychiatric hospitalization and the timing of stroke. Furthermore, in our study, we considered personal medical history as a possible risk factor for ischemic stroke looking into the types of medical drugs used to treat these medical illnesses, which could also be a possible risk factor for stroke, as well as participant adherence to these drugs. Another possible limitation of the study is the limited number of individuals who have been exposed to schizophrenia and BD in the two groups, which might possibly influence their substantial connection with ischemic stroke, which may have resulted in information bias. It is well known that psychiatric conditions can be challenging to recognize, misdiagnose, or undertreat, and some healthcare providers may fail to recognize psychiatric disorders, so even if caregivers were close to the stroke patient, some of them may have been unaware of these private conditions or may have provided sociably desirable answers. Furthermore, the patients and controls who were questioned may have been in denial or provided socially acceptable responses. As a result, more research is needed to improve the quality of data regarding the link between schizophrenia, BD, AUD, and stroke risk by engaging psychiatrists in the data collection process to further validate the psychiatric diagnosis, particularly for participants who attempted to conceal their clinical diagnosis.

Another potential disadvantage is the possibility of selection bias. As in any volunteer research, individuals who refused to participate may have characteristics comparable to the general population; particularly, it generates a sample of controls that may not be typical of general population exposure. Additionally, when participants were questioned about the reasons for their rejection or even partial replies, it was mostly owing to the fact that psychiatric diseases are an embarrassing and private issue.

Amidst these drawbacks, this study adds to the existing literature on ischemic stroke risk factors, notably schizophrenia, BD, and AUD, and sheds light on which mental illnesses were more strongly associated with stroke risk and severity. Furthermore, we were able to limit the chance of missing patients’ medical and psychiatric premorbidity by utilizing our standardized questionnaire to assess independent variables, such as schizophrenia, BD, and AUD. Although this is an observational study, it suggests that people with effective disorder should be given greater attention to their physical health and that they should receive additional follow-up from their physicians.

## 6. Clinical Implications

We believe that there is an urgent need to initiate research attempts to gain a better understanding of the underlying pathophysiological mechanisms of stroke and their association with schizophrenia, BD, and AUD; thus, future studies should primarily incorporate a longitudinal cohort design with adequate follow-up periods and rigorous monitoring of exposure and outcomes. It’s a good idea to schedule standardized follow-up assessments after 7 and 90 days to evaluate the long-term result. Moreover, whether our findings are connected to the natural history of schizophrenia or BD, treatments for schizophrenia and BD will need to be investigated further in future studies. 

Further, our data clearly imply that reducing alcohol intake among heavy drinkers is a significant approach to stroke prevention in the general population. Based on our findings, we believe that more progress needs to be made in raising public awareness to support healthy lifestyle behavior and limit the uptake of harmful health practices because other factors (such as unhealthy lifestyles, poor healthcare, and so on) may also contribute to the increased events of stroke in schizophrenia, BD, and AUD patients. 

Nonetheless, our study highlights the need for healthcare providers to be on the alert for signs of a potential psychiatric disorder in their patients, particularly those at high risk of stroke, and to investigate whether prompt intervention for psychiatric disorders can reduce the risk of ischemic stroke. Additionally, since stroke severity was found to be highly associated with a history of schizophrenia, BD, and AUD, it is crucial that clinicians are aware of these potential risk factors and take them into account when designing programs to enhance outcomes after stroke by incorporating attempts to screen at-risk patients for post-stroke severe symptoms, initiating treatment, and establishing more effective strategies to encourage rehabilitation participation and secondary prevention compliance.

Finally, the benefits of exercise should not be questioned but rather reinforced. However, more research is needed to examine the impact of different levels of physical activity at different ages, together with medical comorbidities, on the risk of developing stroke.

## 7. Conclusions

The findings of our study suggest that individuals with schizophrenia, BD, and AUD-related disorders may be at a higher risk for ischemic stroke and exhibit more severe symptoms. These are intriguing points to consider in future research, which could lead to a better understanding of the relationship between the neurological basis of these disorders and the risk and severity of ischemic stroke, paving the way for treatment development. We believe that the first step toward creating beneficial preventative and treatment interventions is determining individuals with schizophrenia, BD, or AUD, assessing their risk of ischemic stroke, and developing more integrated treatments. Furthermore, it is essential to emphasize the necessity of physicians closely monitoring the long-term outcome of stroke patients with co-occurring schizophrenia, BD, and AUD. 

To avoid any negative outcomes, practicing psychiatrists and other healthcare professionals must be informed of and encouraged to be aware of the positive effects of psychomotor and robot therapy on cognitive functions, independence, social adaptability, and somatic changes in patients with severe illnesses, such as ischemic stroke as demonstrated by various studies [84,85]. The scientific and healthcare communities are then asked to assist the country’s mental health sector by incorporating new and innovative technologies to assist these patients. This will contribute to the reduction of unintended outcomes, such as ischemic stroke. 

## Figures and Tables

**Table 1:** Bivariate analysis of demographic factors associated with ischemic stroke.

Variable	Ischemic Stroke Patients (N = 113)	Ischemic Stroke-Free Patients (N = 451)	*p*-Value
	**Mean ± SD**		**0.035**
**Age**	65.5 ± 11.9	62.9 ± 11.6	
**Gender**	**N (%)**		1
Male	51 (45.1%)	203 (45.0%)	
Female	62 (54.9%)	248 (55.0%)	
**Marital Status**			**0.020**
Single	13 (11.5%)	135 (29.9%)	
Married	85 (75.2%)	286 (63.4%)	
Divorced	3 (2.7%)	20 (4.4%)	
Widowed	12 (10.6%)	10 (2.2%)	
**Educational Level**			**<0.001**
Primary-Complementary	61 (54.0%)	141 (31.3%)	
Secondary	20 (17.7%)	164 (36.4%)	
University	32 (28.3%)	146 (32.4%)	
**Monthly Income**			**<0.001**
Low (<1000 USD)	66 (58.4%)	290 (64.3%)	
Intermediate (1000–2000 USD)	25 (22.1%)	147 (32.6%)	
High (>2000 USD)	22 (19.5%)	14 (3.1%)	
**Preexisting Physical Disorders**			
Hypertension	82 (72.6%)	254 (56.3%)	**0.002**
Dyslipidemia	65 (57.5%)	205 (45.5%)	**0.027**
Diabetes	41 (36.3%)	120 (26.6%)	**0.048**
Heart Diseases	48 (42.5%)	53 (11.8%)	**<0.001**
Atrial Fibrillation	34 (30.1%)	36 (8.0%)	**<0.001**
Asthma-COPD	44 (38.9%)	118 (26.2%)	**0.008**
Cancer	2 (1.8%)	15 (3.3%)	0.545
Obesity	70 (61.9%)	229 (50.8%)	**0.035**
	**Mean ± SD**		
**Physical Activity (IPAQ)**	163.83 ± 232.74	79.28 ± 139.31	**<0.001**
**Mediterranean Diet Adherence** **(MeD)**	5.4 ± 3.1	7.6 ± 1.9	**<0.001**

The variables that are not present in the table did not show a significant association with ischemic stroke. Numbers in bold indicate significant *p*-values.

**Table 2:** Bivariate analysis of other factors associated with ischemic stroke.

Variable	Presence of Ischemic Stroke (N = 113)	Absence of Ischemic Stroke (N = 451)	*p*-Value
	**Mean ± SD**		
**AUD**	3.8 ± 2.7	3.1 ± 1.8	**0.014**
**BD**	2.6 ± 2.8	1.8 ± 1.9	**0.003**
	**N (%)**		
**Schizophrenia**	4 (3.5%)	3 (0.7%)	**0.033**

BD = bipolar disorder, AUD = alcohol use disorder. Numbers in bold indicate significant *p*-values.

**Table 3:** Adjusted odds ratios with their 95% confidence intervals from the logistic regression of ischemic stroke among cases and control.

Logistic Regression Taking the Presence vs. Absence of Ischemic Stroke as the Dependent Variable and Taking AUD, BD, and Schizophrenia as Independent Variables.			
**Variables**	***p***	**aOR**	**95% CI**
**Schizophrenia**	**0.035**	6.162	1.136–33.423
**BD**	**0.025**	4.653	1.214–17.834
**AUD**	**0.003**	3.918	1.584–9.689
**Obesity**	**0.032**	1.732	1.049–2.861
**Physical Activity (IPAQ)**	**<0.001**	4.614	2.669–7.978
**Atrial Fibrillation**	**0.010**	2.415	1.235–4.721
**Diabetes**	**0.017**	1.865	1.117–3.115
**Heart Diseases**	**<0.001**	9.890	5.099–19.184
**Asthma-COPD**	**0.008**	1.971	1.190–3.263

Backward stepwise likelihood ratio method; logistic regression. Variables entered: age, marital status, educational level, hypertension, dyslipidemia, diabetes, heart diseases, atrial fibrillation, asthma-COPD, obesity, Mediterranean diet adherence, physical activity, schizophrenia, bipolar disorder (BD), alcohol use disorder (AUD). aOR: adjusted odds ration; CI: 95% confidence interval; numbers in bold indicate significant *p*-values.

**Table 4:** Bivariate analysis of variables associated with the levels of stroke severity.

Variable	Levels of Stroke Severity			*p*-Value
	No Stroke	Minor/Moderate Stroke	Moderate to Severe/Severe Stroke	
	**Mean ± SD**			**<0.001**
**Age**	62.88 ± 11.6	59.40 ± 10.7	75.00 ± 6.2	
**Gender**	**N (%)**			0.772
Male	203 (45.0%)	33 (47.8%)	18 (40.9%)	
Female	248 (55.0%)	36 (52.2%)	26 (59.1%)	
**Marital Status**				**0.027**
Single/Divorced/Widowed	165 (36.6%)	14 (20.3%)	14 (31.8%)	
Married	286 (63.4%)	55 (79.7%)	30 (68.2%)	
**Educational Level**				**<0.001**
Primary-Complementary	141 (31.3%)	29 (42.0%)	32 (72.7%)	
Secondary	164 (36.4%)	9 (13.0%)	11 (25.0%)	
University	146 (32.4%)	31 (44.9%)	1 (2.3%)	
**Preexisting Physical Disorders**				
Hypertension	254 (56.3%)	49 (71.0%)	33 (75.0%)	0.006
Dyslipidemia	205 (45.5%)	36 (52.2%)	29 (65.9%)	0.025
Diabetes	120 (26.6%)	24 (34.8%)	17 (38.6%)	0.109
Heart Diseases	53 (11.8%)	19 (27.5%)	29 (65.9%)	<0.001
Atrial Fibrillation	36 (8.0%)	16 (23.2%)	18 (40.9%)	<0.001
Asthma-COPD	118 (26.2%)	25 (36.2%)	19 (43.2%)	0.019
Cancer	15 (3.3%)	1 (1.4%)	1 (2.3%)	0.744
Obesity	229 (50.8%)	43 (62.3%)	27 (61.4%)	0.105
	**Mean ± SD**			
**Physical Activity (IPAQ)**	79.28 ± 139.31	253.95 ± 251.37	22.50 ± 87.68	**<0.001**
**Mediterranean Diet Adherence**	7.60 ± 1.9	5.50 ± 3.3	5.20 ± 2.9	**<0.001**
**Preexisting Psychological Disorders**				
AUD	1.42 ± 1.9	3.38 ± 3.2	2.70 ± 2.0	**<0.001**
BD	1.78 ± 1.9	3.25 ± 3.2	1.68 ± 1.7	**<0.001**
	**N (%)**			**0.039**
Schizophrenia	3 (0.07%)	2 (2.9%)	2 (4.5%)	

Numbers in bold indicate significant *p*-values.

**Table 5:** Multivariable analysis: multinomial regression taking the levels of stroke severity.

Model 1: Levels of Stroke Severity (Minor/Moderate Stroke vs. No Stroke)			
Variables	*p*	aOR	95% CI
Age	0.415	0.978	0.923–1.032
Marital Status (married vs single *)	0.078	2.121	0.920–4.893
Educational Level (secondary vs primary-complementary *)	0.187	0.519	0.196–1.374
Educational Level (university vs primary-complementary *)	**<0.001**	0.130	0.044–0.383
AUD	**<0.001**	1.466	1.260–1.706
BD	**0.004**	1.229	1.066–1.416
Schizophrenia	0.620	2.093	0.113–38.771
Physical Activity (IPAQ)	**<0.001**	1.006	1.004–1.008
Mediterranean Diet Adherence	**<0.001**	0.711	0.620–0.816
Hypertension (yes vs. no *)	**0.037**	2.380	1.055–5.372
Dyslipidemia (yes vs. no *)	**0.043**	2.220	1.024–4.811
Heart Diseases (yes vs. no *)	**0.009**	3.726	1.396–9.950
Atrial Fibrillation (yes vs. no *)	0.106	2.193	0.846–5.685
Asthma-COPD (yes vs. no *)	0.136	1.708	0.845–3.452
**Model 2: Levels of stroke severity (Moderate to Severe/Severe Stroke vs No Stroke)**			
**Variables**	**p**	**aOR**	**95% CI**
Age	**<0.001**	1.321	1.173–1.488
Marital Status (married vs single *)	0.383	1.577	0.567–4.385
Educational Level (secondary vs primary-complementary *)	0.590	1.333	0.413–4.298
Educational Level (university vs primary-complementary *)	0.630	0.109	0.009–1.267
AUD	**<0.001**	1.719	1.385–2.133
BD	**<0.001**	1.656	1.281–2.141
Schizophrenia	**0.006**	6.884	3.294–11.492
Physical Activity (IPAQ)	0.663	1.001	0.995–1.008
Mediterranean Diet Adherence	**<0.001**	0.573	0.470–0.698
Hypertension (yes vs. no *)	0.411	0.636	0.216–1.872
Dyslipidemia (yes vs. no *)	0.907	0.945	0.363–2.460
Heart Diseases (yes vs. no *)	**0.002**	6.486	1.955–21.525
Atrial Fibrillation (yes vs. no *)	0.271	1.788	0.636–5.031
Asthma-COPD (yes vs. no *)	0.134	1.958	0.813–4.719

* Reference group; aOR: adjusted odds ratio; CI: 95% confidence interval; numbers in bold indicate significant *p*-values.

## Data Availability

The data presented in this study are available on request from the corresponding author. The data are not publicly available due to the rules of the ethics committee.

## Abbreviations

BD	Bipolar Disorder
AUD	Alcohol Use Disorder
MeD	Mediterranean Diet
SPSS	Statistical Package for Social Science

## Appendix A

### Questionnaire Form: English Version

*This study was conducted in Lebanon to determine if preexisting mental disorders can increase a patient’s future risk of ischemic stroke. This research is entirely voluntary, and the following questionnaire is available in Arabic. We kindly request that you respond credibly to the questions posed in the statistics. We promise to maintain strict confidentiality and only use the information for scientific purposes.*

I. Socio-Demographic Information:
  1. Gender: □ M □ F
  2. Age: ………….
  3. Weight: ……….
  4. Height: …………
  5. Marital status: □ Single □ Married □ Divorced □ Widower
  6. Educational Level: □ Elementary □ Intermediate □ Secondary □ University □ Illiterate
  7. Work Status: □ Works □ Jobless □ Retired/Housewife
  8. Monthly Income: □ <USD 1000 □ USD 1000–2000 □ USD >2000
  9. Health Coverage: □ Social Security □ Personal Medical Insurance □ State Employees Cooperative □ Personal Account
  10. Region: □ Beirut □ South □ Mount Lebanon □ Bekaa □ North
II. Personal Clinical Factors
  1. Has a “physician” ever diagnosed you with:
    - Hypertension:
      □ Yes □ No □ I don’t know/I’m not sure
    - Dyslipidemia:
      □ Yes □ No □ I don’t know/I’m not sure
    - Diabetes:
      □ Yes □ No □ I don’t know/I’m not sure
    - Any type of heart disease:
      □ Yes □ No □ I don’t know/I’m not sure
    - Atrial fibrillation:
      □ Yes □ No □ I don’t know/I’m not sure
    - Respiratory problem (Specifically: Asthma, COPD):
      □ Yes □ No □ I don’t know/I’m not sure
    - Cancer:
      □ Yes □ No □ I don’t know/I’m not sure
    - Stroke:
      □ Yes □ No □ I don’t know/I’m not sure
      If YES:
      h.1.Date of Stroke: From _____ week(s)
      h.2. Stroke Type:
      □ Ischemic stroke
      □ Hemorrhagic stroke
      □ Transient ischemic attack
      h.3. Diagnosis:
      □ Complete blood count
      □ Clotting time PT (prothrombin time) and PTT (partial thromboplastin time)
      □ Computerized tomography (CT) scan
      □ Magnetic resonance imaging (MRI)
      □ Carotid ultrasound
      □ Echocardiogram
      □ Transient ischemic attack
      □ Angiograms of your head and neck
      h.4. Stroke Severity: National Institutes of Health Stroke Scale (NIHSS):
      □ 0 = No stroke
      □ 1–4 = Minor stroke
      □ 5–15 = Moderate stroke
      □ 16–20 = Moderate to Severe stroke
      □ 21–42 = Severe stroke
      h.5. Time of Hospital Stay (if possible): _____ days
      h.6. Stroke Complications:
      □ Paralysis or loss of ability to move muscles
      □ Difficulty speaking or swallowing
      □ Memory loss or problems with general comprehension
      □ Aches
  2. Questionnaire of Mediterranean Diet Adherence
    	Yes	No
    1.Is olive oil the main culinary fat used?		
    2. Are ≥ 4 tablespoons of olive oil used each day?		
    3. Are ≥ 2 servings (of 200 g each) of vegetables eaten each day?		
    4. Are ≥ 3 servings of fruit (of 80 g each) eaten each day?		
    5. Is < 1 serving (100–150 g) of red meat/hamburgers/other meat products eaten each day?		
    6. Is < 1 serving (12 g) of butter, margarine or cream eaten each day?		
    7. Is < 1 serving (330 mL) of sweet or sugar sweetened carbonated beverages consumed each day?		
    8. Are ≥ 3 glasses (of 125 mL) of wine consumed each week?		
    9. Are ≥ 3 servings (of 150 g) of legumes consumed each week?		
    10. Are ≥ 3 servings of fish (100–150 g) or seafood (200 g) eaten each week?		
    11. Are < 3 servings of commercial sweets/pastries eaten each week?		
    12. Is ≥ 1 serving (of 30 g) of nuts consumed each week?		
    13. Is chicken, turkey, or rabbit routinely eaten instead of veal, pork, hamburger, or sausage?		
    14. Are pasta, vegetables, or rice dishes flavoured with garlic, tomato, leek, or onion eaten ≥ twice a week?		
    (a) Have you exercised or been involved in sports or physical activity in the past 12 months?
      □ Y □ N
    (b) If your answer is yes to question a, what is the intensity of physical activity?
      - Continuous heavy breathing with perspiration b. Heavy intermittent breathing with perspiration (tennis, basketball) c. Moderate to severe (recreational sports, cycling) d. Moderate (volleyball) e. Mild (fishing, walking).
  3. International Physical Activity Questionnaire (IPAQ)
    We are interested in finding out about the kinds of physical activities that you do as part of your everyday life. The questions will ask you about the time you spent being physically active in the last 7 days. Please answer each question even if you do not consider yourself to be an active person.
    1. During the last 7 days, on how many days did you do vigorous physical activities, such as heavy lifting, digging, aerobics, or fast bicycling?
      _____ days per week
      □ No vigorous physical activities → Skip to question 3
    2. How much time did you spend doing vigorous physical activities on one of those days?
      _____ hours per day _____ minutes per day
      □ Don’t know/Not sure
    3. During the last 7 days, on how many days did you do moderate physical activities, such as carrying light loads, bicycling at a regular pace, or doubles tennis? Do not include walking.
      _____ days per week
      □ No moderate physical activities → Skip to question 5
    4. How much time did you spend doing moderate physical activities on one of those days?
      _____ hours per day _____ minutes per day
      □ Don’t know/Not sure
    5. During the last 7 days, on how many days did you walk for at least 10 min at a time?
      _____ days per week
      □ No walking → Skip to question 7
    6. How much time did you spend walking on one of those days?
      _____ hours per day _____ minutes per day
      □ Don’t know/Not sure
    7. During the last 7 days, how much time did you spend sitting on a week day?
      _____ hours per day _____ minutes per day
      □ Don’t know/Not sure
III. Assessment of Mental Health
  - Do you have a psychiatrist diagnosis of:
  □ Alcohol Use Disorder □ Bipolar Disorder □ Schizophrenia
  - OR how often have you been bothered by any of the following problems?
  1. The Alcohol Use Disorders Identification Test (AUDIT)
    How often do you have a drink containing alcohol?□ Never □ Monthly or less □ 2–4 times a month □ 2–3 times a week □ 4 or more times a week
    How many standard drinks containing alcohol do you have on a typical day when drinking? □ 1 or 2 □ 3 or 4 □ 5 or 6 □ 7 to 9 □ 10 or more
    How often do you have six or more drinks on one occasion?□ Never □ Less than monthly □ Monthly □ Weekly □ Daily or almost daily
    During the past year, how often have you found that you were not able to stop drinking once you had started?□ Never □ Less than monthly □ Monthly □ Weekly □ Daily or almost daily
    During the past year, how often have you failed to do what was normally expected of you because of drinking?□ Never □ Less than monthly □ Monthly □ Weekly □ Daily or almost daily
    During the past year, how often have you needed a drink in the morning to get yourself going after a heavy drinking session?□ Never □ Less than monthly □ Monthly □ Weekly □ Daily or almost daily
    During the past year, how often have you had a feeling of guilt or remorse after drinking?□ Never □ Less than monthly □ Monthly □ Weekly □ Daily or almost daily
    During the past year, have you been unable to remember what happened the night before because you had been drinking?□ Never □ Less than monthly □ Monthly □ Weekly □ Daily or almost daily
    Have you or someone else been injured as a result of your drinking?□ No □ Yes, but not in the past year □ Yes, during the past year
    Has a relative or friend, doctor, or other health worker been concerned about yourdrinking or suggested you cut down?□ No □ Yes, but not in the past year □ Yes, during the past year
  2. The Mood Disorder Questionnaire (MDQ)
    1.Has there ever been a period of time when you were not your usual self and	Yes	No
      you felt so good or so hyper that other people thought you were not your normal self, or you were so hyper that you got into trouble?		
      you were so irritable that you shouted at people or started fights or arguments?		
      you felt much more self-confident than usual?		
      you had much less sleep than usual and found you didn’t really miss it?		
      you were much more talkative or spoke much faster than usual?		
      thoughts raced through your head or you couldn’t slow your mind down?		
      you were so easily distracted by things around you that you had trouble concentrating or staying on track?		
      you had much more energy than usual?		
      you were much more active or did many more things than usual?		
      you were much more social or outgoing than usual, for example, you telephoned friends in the middle of the night?		
      you were much more interested in sex than usual?		
      you did things that were unusual for you or that other people might have thought were excessive, foolish, or risky?		
      spending money got you or your family into trouble?		
    2. If you checked YES to more than one of the above, have several of these ever happened during the same period of time?
      □ Yes □ No
    3. How much of a problem did any of these cause you, such as being unable to work; having family, money or legal troubles; getting into arguments or fights? Please circle one response only.
      □ No Problem □ Minor Problem □ Moderate Problem □ Serious Problem
    4. Have any of your blood relatives (i.e., children, siblings, parents, grandparents, aunts, uncles) had manic-depressive illness or bipolar disorder?
      □ Yes □ No
    5. Has a health professional ever told you that you have manic-depressive illness or bipolar disorder?
      □ Yes □ No
  3. Mini International Neuropsychiatric Interview (MINI) for Psychotic Disorders
    Ask for an example of each question answered positively. Code yes only if the examples clearly show a distortion of thought or of perception or if they are not culturally appropriate.
    Now I am going to ask you about unusual experiences that some people have.
    1. (a) Have you ever believed that people were spying on you, or that someone was plotting against you, or trying to hurt you? □ No □ Yes
      (b) IF YES: do you currently believe these things?
    2. (a) Have you ever believed that someone was reading your mind or could hear your thoughts, or that you could actually read someone’s mind or hear what another person was thinking? □ No □ Yes
      (b) IF YES: do you currently believe these things?
    3. (a) Have you ever believed that someone or some force outside of yourself put thoughts in your mind that were not your own, or made you act in a way that was not your usual self? Have you ever felt that you were possessed? □ No □ Yes
      (b) IF YES: do you currently believe these things?
    4. (a) Have you ever believed that you were being sent special messages through the TV, radio, internet, newspapers, books, or magazines or that a person you did not personally know was particularly interested in you? □ No □ Yes
      (b) IF YES: do you currently believe these things?
    5. (a) Have your relatives or friends ever considered any of your beliefs odd or unusual? □ No □ Yes
      (b) IF YES: do they currently consider your beliefs strange or unusual?
    6. (a) Have you ever heard things other people couldn’t hear, such as voices? □ No □ Yes
      (b) IF YES TO VOICE HALLUCINATION: Was the voice commenting on your thoughts or behavior or did you hear two or more voices talking to each other?
      (a) IF YES: have you heard sounds/voices in the past month?
      (b) IF YES: Was the voice commenting on your thoughts or behavior or did you hear two or more voices talking to each other? □ No □ Yes
    7. (a) Have you ever had visions when you were awake or have you ever seen things other people couldn’t see? □ No □ Yes
      (b) IF YES: have you seen these things in the past month? □ No □ Yes
      CLINICIAN’S JUDGMENT:
    8. (a) Did the patient ever in the past exhibit disorganized, incoherent or derailed speech, or marked loosening of associations? □ No □ Yes
      (b) Is the patient currently exhibiting incoherence, disorganized or derailed speech, or marked loosening of associations? □ No □ Yes
    9. (a) Did the patient ever in the past exhibit disorganized or catatonic behavior? □ No □ Yes
      (b) Is the patient currently exhibiting disorganized or catatonic behavior? □ No □ Yes
    10. (a) Did the patient ever in the past have negative symptoms, e.g., significant reduction of emotional expression or affective flattening, poverty of speech (alogia) or an inability to initiate or persist in goal-directed activities (avolition)? □ No □ Yes
      (b) Are negative symptoms of schizophrenia, e.g., significant reduction of emotional expression or affective flattening, poverty of speech (alogia), or an inability to initiate or persist in goal-directed activities (avolition), prominent during the interview?
      □ No □ Yes
    11. (a) Are 1 or more “a” questions from 1a to 7a, coded YES?
      And is either:
      MAJOR DEPRESSIVE EPISODE, (current, recurrent, or past)
      OR
      MANIC OR HYPOMANIC EPISODE, (current or past) coded YES?
      AND
      How long has the mood episode lasted? _______________
      How long has the psychotic episode lasted? _______________
      If such a mood episode is present, code YES to 11a only if the mood disturbance is present for the majority of the total duration of the active and residual periods of the psychotic symptoms. Otherwise, code NO.
      □ No □ Yes
